# Characterizing the Personalized Microbiota Dynamics for Disease Classification by Individual-Specific Edge-Network Analysis

**DOI:** 10.3389/fgene.2019.00283

**Published:** 2019-04-11

**Authors:** Xiangtian Yu, Xiaoyu Chen, Zhenjia Wang

**Affiliations:** ^1^Shanghai Jiao Tong University Affiliated Sixth People’s Hospital, Shanghai, China; ^2^Center for Public Health Genomics, University of Virginia, Charlottesville, VA, United States

**Keywords:** network, individual-specific edge-network analysis, complex diseases, personalized microbiota dynamics, omics data

## Abstract

Environmental factors such as the gut microbiome are thought to play an important role in the development and treatment of many diseases. But our understanding of microbiota compositional dynamics is still unclear and incomplete because the intestinal microbial community is an easily-changed ecosystem. It is urgently required to understand the large variations among individuals. These variations, however, will be an asset rather than a limitation to personalized medicine. For a proof-of-concept study on individual-specific disease classification based on microbiota compositional dynamics, we implemented an adjusted individual-specific edge-network analysis (iENA) method to address a limited number of samples from one individual, and compared it to the temporal 16S rRNA (ribosomal RNA) gene sequencing data from individuals in a challenge study. Our identified individual-specific OTU markers or their combined markers are consistent with previously reported markers, and the predictive score based on them can perform a better AUROC than the previous 0.83 and achieve about 90% accuracy on predicting whether an individual developed diarrhea [i.e., were symptomatic (Sx)] or not. In addition, iENA also showed satisfactory efficiency on another dataset about bacterial vaginosis (BV). All these results suggest that the combination of high-throughput microbiome experiments and computational systems biology approaches can efficiently recommend potential candidate species in the defense against various pathogens for precision medicine.

## Introduction

In addition to genetic risks, environmental factors are accumulating more and more evidence regarding their critical roles in human complex diseases (Qin et al., 2012; Hoyles et al., 2018). As one of these key factors, the gut microbiome is gradually being accepted to be a key player in controlling disease development and progression (Claesson et al., 2012; Forslund et al., 2015). Many studies have concluded that the alterations of commensal microbiota may contribute to a range of significant pathogen states such as antibiotic-associated diarrhea, inflammatory bowel disease, irritable bowel syndrome, pseudomembranous colitis, and cancer (Pop et al., 2016). The high-throughput sequencing of microbial communities provides a bio-technical foundation to characterize the associations of the host microbiome (Blow, 2008; Pushkarev et al., 2018), which is helpful to detect pathogens and identify the crosstalk between an organism’s microbiome and the environment (Wagner et al., 2018). This frontier research not only links intestinal microbial communities with health or disease phenotypes but also provides lots of processed data for public requirement and reuse.

As is known, the intestinal microbial community is actually a more complex ecosystem with essential influences on host health status in numerous ways, such as regulating metabolism, developing immunity, and suppressing enteropathogens (Gill et al., 2006; Round and Mazmanian, 2009; Maynard et al., 2012). These beneficial co-evolved interactions between host and microbiota can be disrupted by different environmental stresses such as changes in dietary habits, natural physiology, virus infections, and medical treatments (Dethlefsen et al., 2008; Wu et al., 2011; Pop et al., 2014). Specifically, antibiotic treatments for enteric infections such as ETEC may even lead to immediate and significant changes of gut microbiota (Dethlefsen and Relman, 2011), e.g., loss of beneficial species, increase of drug-resistant strains, and predisposition of pathogen infections. The intestinal ecosystem is easily changed, although it is able to recover and is often incomplete (Lozupone et al., 2012). Thus, it is necessary to carry on long-term observational studies to detect the possible permanent functional alterations among certain microbiota (Jernberg et al., 2010).

Despite the critical role of microbiota in human health attracting more attention, our understanding of microbiota compositional dynamics is still incomplete, and more well-designed analytical methods are also required to make full use of rich data resources. In the gradually increasing observational studies of the gut microbiota, the microbial communities’ sequencing data, e.g., metagenomics data, are widely tested and analyzed (Vedoy et al., 2018). Different from the other high-throughput data in genetic studies (Yu and Zeng, 2018), metagenomics data can be easily changed within different conditions and individuals. Thus, individual heterogeneity is particularly important and individual variation should not be ignored in analytical approaches. In fact, in the era of precision medicine, many methods have focused on the common molecular biomarkers which can diagnose disease states at the cohort/population level. However, to study the occurrence and progression of a disease in a given patient (Zeng et al., 2014; Yu et al., 2015), accurate diagnosis of individuals by sample-specific biomarkers is a key concept and action (Zeng et al., 2016). In contrast to the traditional molecular biomarker analysis, our previously proposed individual-specific edge-network analysis (iENA) (Yu et al., 2017) combined with dynamic network biomarker (DNB) (Li et al., 2014) has detected the early-warning signals or the pre-disease state before serious disease deterioration based on second-order statistics from the observed data, e.g., “covariance” for expressions among genes or proteins.

Holding an assumption that the microbiota like genetic molecules will have significant network characteristics associated with phenotypes (Rakoff-Nahoum et al., 2016), it is worth extracting discriminative and interpretative features from the microbiota community-like gene network to monitor the disruption of microbial communities during disease occurrence and development (Wang et al., 2018). To take a proof-of-concept study on the dynamic change of intestinal ecosystem and their disease signals, we have adjusted the iENA method (Yu et al., 2017) with reference group to address the limited number of samples from one individual, and applied it to analyze temporal high-throughput 16S rRNA data from individuals, which is expected to overcome the great individual difference and changeability of the intestinal ecosystem and reveal biological and biomedical insights.

To carry out a proof-of-concept study on the individual-specific disease classification based on microbiota compositional dynamics, we captured the temporal changes from microbiota data of volunteers during the ETEC challenge and subsequent treatment with ciprofloxacin (Pop et al., 2016), and we found the following: (i) the common microbiota biomarkers (OTUs) reported in the previous work can be mostly recovered and are also more effective in distinguishing clinical phenotypes of individuals; (ii) individual-specific biomarkers can be detected depending on the temporal 16S rRNA data from each subject and the given reference data from multiple subjects; (iii) the individual microbiota data can be used to effectively carry out statistics, explore and integrate for personalized diagnosis, prognosis and prediction. In addition, in order to further validate the efficacy and robustness of our concept and method, we have employed iENA on another real-world data from the daily composition and relative abundance of bacteria in vaginal samples from twenty-five women with and without bacterial vaginosis (BV), and again satisfactory performance was achieved on distinguishing BV occurrence from healthy controls. In total, this work supplied novel evidence of individual biomarkers to promoting microbiota-based disease classification, while the high-ranked critical OTUs deserve future clinical validations.

## Materials and Methods

### Description of Data Organization Used in Proof-of-Concept Study

*Escherichia coli* (ETEC) has two expected outcomes: watery diarrhea as symptomatic (Sx), or the host remains asymptomatic (Asx) (Pop et al., 2016). The wild-type virulent ETEC strain (*E. coli* O78:H11) was most frequently used in volunteer studies, which induces severe diarrhea, with mild fever and vomiting being reported in a relatively higher proportion of subjects. The 16S rRNA data from gut microbiota reported in previous volunteer challenge studies with ETEC H10407 were selected for our study (Pop et al., 2016), which can be obtained from NCBI under project ID: PRJNA298336. The simple summary of the challenge protocol are as follows: the health status of subjects in this volunteer challenge was assessed before the challenge; early antibiotic treatment was given to the patients when some symptoms appear; and starting on day 5, all subjects received a 3-day ciprofloxacin treatment. Importantly, the stool specimens were collected at 12 time points: prior to ETEC infection (day −1, 0) and on days 1–7, 9, 28, and 84 after the infection (Pop et al., 2016). After sequencing, 124 samples finally passed quality controls and time matches which were used in our analysis, corresponding to 50 samples from 5 Sx volunteers and 74 samples from 7 Asx volunteers (Pop et al., 2016).

### Temporal Microbiota Data Analysis by Individual-Specific Edge-Network Analysis (iENA)

**FIGURE 1:**
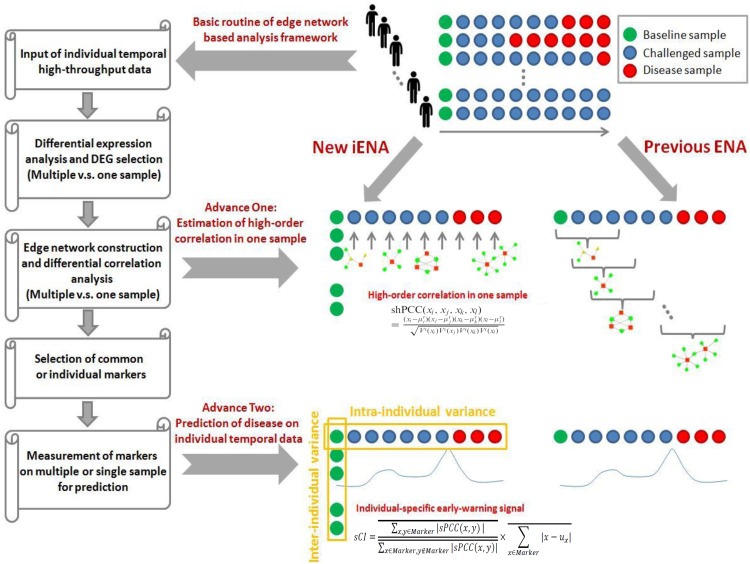
Concept of edge-network and individual-specific edge-network analysis (iENA).

We previously developed an advanced computational framework, i.e., iENA, based on our proposed high-order correlation measurement as shPCC for one-sample omics data (Yu et al., 2017). In brief, iENA provides a powerful network-analysis tool for studying temporal omics data of complex diseases in a manner of individual samples, which is suitable for applications in precision medicine or personalized medicine. As noted in previous iENA analysis, each individual used some samples in the early stages as network references in dynamics analysis. However, when the number of samples for each individual is limited, this strategy cannot work. Thus, to investigate the microbiota dynamics in this work, we implemented an adjusted iENA particularly using samples from the baseline of individuals as the network reference and applied it for analyzing the temporal 16S rRNA data (or even other metagenomics data) as in Figure 1.

#### Collecting Data

To apply iENA, we downloaded temporal 16S rRNA datasets from NCBI, which include the ETEC challenge infection samples on individual subjects.

#### Selecting Reference Samples

In order to obtain the mean and variance of microbiota compositions used for evaluating each new single sample (i.e., for each sample of one subject at one time point), a group of reference samples (i.e., control samples, or normal samples) is required to be confirmed ahead of follow-up analysis. Here, we set the samples from the normal stage, i.e., the samples at baseline as a reference group. Whether these samples came from the same subject or different subjects are depending on the data organization. Any sample with similar properties could serve as a reference group in theory.

#### Selecting OTUs Based on Non-zero Value

One difficulty for processing 16S rRNA data is to deal with the large number of zero values for iENA, e.g., during any division computation; thus, similar to previous studies, we deleted OTUs with a large percent of zero values (i.e., 85% or other percent determined by a given threshold) to reduce the bias impact.

#### Constructing Microbiota Network by sPCC Calculation

When we had reference samples, we were able to construct the co-expression network of one sample by our single-sample measurement of the Pearson correlation coefficient (sPCC), consistent with previous studies (Yu et al., 2017). Considering the absence of background network for microbial communities, we selected edges (i.e., one edge represented the association between two microbiota, represented by a pair of OTUs here) from a direct rank cut-off for correlations because the distribution of the new PCC values is not the normal distribution. Then, the top-ranked edges with strong relations were finally selected, which consisted of conventional node-network or microbiota community (Wang et al., 2016; Sung et al., 2017), and were used as the background “nodes” for constructing the following edge-network (e.g., a network of OTU-pairs).

#### Constructing Microbiota-Pair Network by shPCC Calculation

Between two OTU-pairs, we carried out the estimation of the fourth-order single-sample correlation coefficient for each edge-pair (i.e., two OTU-pairs) by shPCC (Yu et al., 2017) for each single-sample (e.g., for each sample of one subject at one time point). Note that, in this step, we actually only computed the correlations between the pre-selected OTU-pairs from the above steps, and thus we could reduce the unnecessary computations drastically. Finally, we obtained the microbiota-pair network model corresponding to each sample at a particular time point, and each subject had personalized features on a time series in the OTU-pair networks.

#### Recognizing Individual OTU-Pair Biomarkers

Similar to the OTU-pair selection, we selected top-ranked edge-pairs as edge-biomarkers (i.e., OTU-pair biomarkers), which have strong relations with each other in terms of the high-order compositional correlations. Those strong correlated OTU-pairs can be viewed as DNB candidates, represented as a set called “Marker.” Then, for each individual, the OTU-pairs in the edge-network were used as individual OTU-pair biomarkers, and these OTUs were applied in the clinical phenotype prediction.

#### Quantifying the Predictive Markers by sCI

As is known, the DNB has been developed to identify the pre-disease state before a sudden deterioration during disease development and progression as general disease-warning signals (Chen et al., 2012; Zeng et al., 2013). Recently, the DNB model with its quantification criterion (i.e., CI, composite index) based on multiple samples has been widely adopted:

(1) $$\mathrm{CI}=\frac{\bar{{\mathrm{PCC}}_{\mathrm{in}}}}{\bar{{\mathrm{PCC}}_{\mathrm{out}}}}\times \bar{{\mathrm{SD}}_{\mathrm{in}}}$$

In our previous work on gene networks, the DNB criterion is further re-defined from the above correlation measurements in a manner of single-sample, i.e., sCI is defined as:

(2) sCI=∑x,y∈Marker|sPCC(x,y)|∑x∈Marker,y∈Marker|sPCC(x,y)|×∑x∈Marker|x−ux|¯

where $\bar{{\mathrm{PCC}}_{\mathrm{in}}}$ is the average PCC of the compositions of OTUs in the dominant group or DNB (e.g., a group of marker OTUs or molecules) in absolute value in one sample; $\bar{{\mathrm{PCC}}_{\mathrm{out}}}$ is the average PCC of the compositions of OTUs between the dominant group and other in absolute value in one sample; $\bar{{\mathrm{SD}}_{\mathrm{in}}}$ is the average standard deviation of the compositions of OTUs in the dominant group or DNB. “Marker” is the set of DNB members. Then, the sCI of individual OTU markers was used to indicate the disease-warning signals when its value was greater than a given threshold.

#### Comparing OTU Markers and Their Disease Classification

For each individual, we obtained the differential OTU-pairs in each single-sample (i.e., the edge associations in each time point) as novel edge-biomarkers to indicate the disease-warning signal. We obtained the sCI value with edge biomarkers for each subject or sample, and we observed different sCI scores at consecutive time points. Thus, the value of sCI changed with time and we defined a threshold to indicate the criticality, i.e., warning disease or not for a subject. In addition, for the challenge data, we also examined the OTU markers induced from each subject, and compared them with previously reported 32-OTU markers from the original research of the experimental data (Pop et al., 2016).

## Results and Discussion

### Parameter Setting of the Analysis on ETEC Challenge Data

To make full use of iENA, we used 16S rRNA (ribosomal RNA) gene sequencing data to describe changes in the fecal microbiota from 12 human volunteers during the challenge study with ETEC (H10407), where three males and two females developed diarrhea symptoms while four males and three females did not (Pop et al., 2016).

**FIGURE 2:**
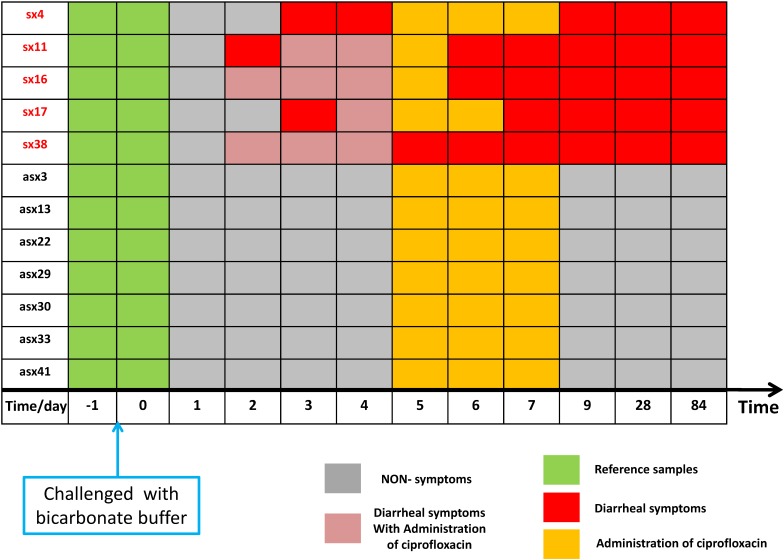
The sample organization of ETEC challenges dataset. The subjects are divided into two groups according to the clinical symptom chart based on standardized symptom scoring: symptomatic (Sx) group with 5 subjects (subjects 4, 11, 16, 17, and 38 in the original data) and asymptomatic (Asx) group with 7 subjects (subjects 3, 13, 22, 29, 30, 33, and 41 in the original data). The samples before the challenge (in green) were used as a reference group; the non-symptom samples (in gray) have no significant clinical symptom; samples in orange indicate administration of ciprofloxacin; red marks represent diarrhea symptoms; pink element indicates the overlapping time/day of diarrhea symptoms and administration of ciprofloxacin.

As shown in Figure 2, according to iENA, we divided subjects into two groups according to clinical symptoms: a Sx group with 5 subjects (subjects 4, 11, 16, 17, and 38 in Figure 2) and an Asx group with 7 subjects (subjects 3, 13, 22, 29, 30, 33, and 41 in Figure 2). Samples before infection from baseline time (green in Figure 2, days −1 and 0) were used as the reference group. After selecting OTUs (non-zero percent > 0.85), we could calculate the sPCC (with mean and variance from the reference group) for each sample. We focused on the edges with strong correlations and finally determined the 1500 strongest relations at each time point. Then, these pre-selected edges were used as the background “nodes” for constructing the edge-network, and the significant signal peaks of edge-biomarkers were captured for each subject across multiple time points, which were candidate DNB members. Different from previous iENA applications, there was another parameter to control; the number of final OTU markers, due to the tested microbiota, was much less than tested human genes or proteins.

### OTU Markers Identified by iENA Are Consistent With Reports in the Literature

Based on the above temporal data, we determined different numbers of OTUs as marking features on each time/sampling point of each subject by iENA, and the OTU-index score (i.e., CI index of OTU markers) is an average measurement against the effect of OTU number. To further prove OTU markers’ satisfactory discrimination of the eventual clinical outcomes of individuals, we identified individual biomarkers comprising differently numbered bacterial OTUs.

**FIGURE 3:**
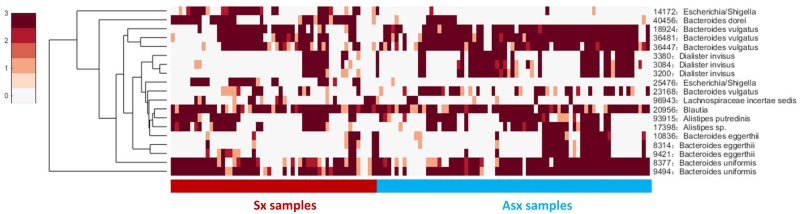
The general abundance of OTU markers in Sx and Asx individuals.

Next, we checked the individual-specific biomarkers by combining all OTUs detected on each sample for the same subject. OTU markers found in five Sx individuals were very different from those identified in Asx individuals, which may be the reason why these OTUs can be used to predict displayed symptoms (or disease occurrence). We finally obtained 19 common OTUs in the Sx group, which were also distinguished from the Asx group in a combination manner (Figure 3).

These 19 common OTU markers represent robust signatures and most of them have been reported in previous works (Pop et al., 2016), which demonstrates the effectiveness of iENA on OTU marker discovery. Patients who eventually developed diarrhea symptoms were primarily affected by the abundance of OTUs from the genus *Bacteroides* as well as *Dialister*. The microbiota predictors included previously observed *Bacteroides* sp., *Blautia* sp., *Alistipes* sp., and our newly found *Escherichia* and *Lachnospiraceae* with a potential role during disease occurrence. Looking at Figure 3 on the one hand, globally, the abundance of OTU signatures seems to be absent in samples of Sx individuals but abundant in samples of Asx individuals; and on the other hand, locally, *Escherichia* and *Lachnospiraceae* appeared most in the samples from Sx individuals. By contrast, some OTUs from *Bacteroides* and *Dialister* are more frequently observed in samples from Asx individuals. These results indicate the biological significance of our OTU markers.

### OTU Markers Outperform Previously Reported OTU Signatures

**FIGURE 4:**
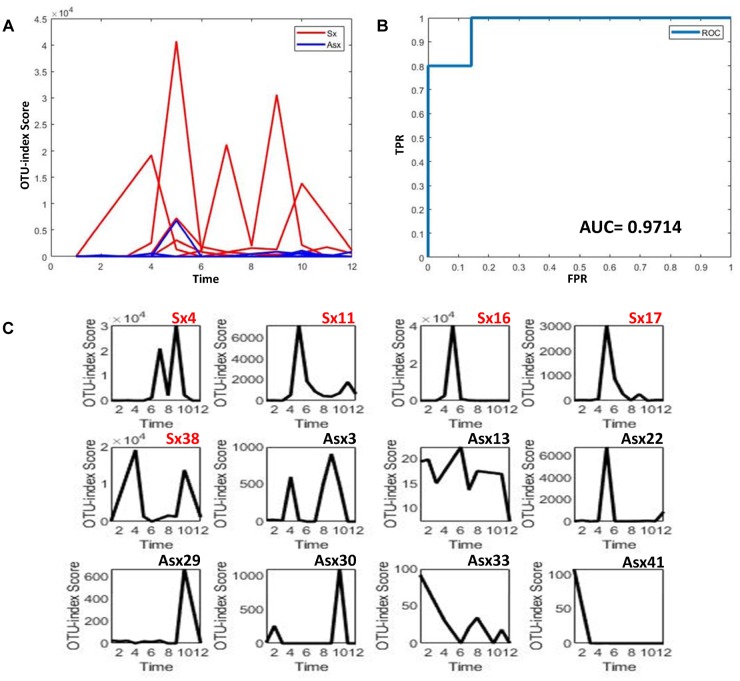
The disease classification performance of OTU markers on the ETEC challenges dataset. **(A)** The predictive score of OTU markers based on CI index from DNB theory. The score curves of subjects from the Sx group are in red, and those of subjects from the Asx group are in blue. Generally, the Sx individuals tend to have a larger score during the disease occurrence. **(B)** The classification evaluation of OTU marker score by ROC and AUC. **(C)** The OTU marker scores correspond to each subject, where the Sx individual will have a large score at earlier time points than Asx individuals.

To further explore whether the microbiota could predict the eventual clinical outcome, we used OTU index scores of above 19 common OTUs to divide individuals into normal and disease groups. With an optimal threshold, the model was able to achieve an AUC of 0.9, larger than previously reported 0.83 (Pop et al., 2016), which means these predictors are robust. Based on these OTUs, the accuracy is about 90% in Figure 4, much larger than the previously reported 76% (Pop et al., 2016), which supports again that the new OTU markers and their quantifications are efficient in judging whether a patient developed diarrhea symptoms or not by individual microbiota data. Following our assumption, the abundance variance rather than abundance level would have more predictive power according to DNB theory, meaning that the OTU-index score of OUT-markers based on abundance variance achieved higher performance.

### Another Case Study on Bacterial Vaginosis (BV)

In order to further validate the efficacy and robustness of our model, we carried out this method on other data (Ravel et al., 2013). This data was obtained from the daily composition and relative abundance of bacteria in vaginal samples from twenty-five women: 15 SBV women diagnosed with Sx BV, six ABV women with Asx BV, and four healthy women at twenty time points during the 10-week study (Ravel et al., 2013). Due to the great influence of bacteria abundance and the association caused by SBV treatment, the bacteria data of the Sx group (9 SBV) and the Asx group (6 ABV and 4 healthy) at the first nine time points ahead of most treatments were used in following analysis.

**FIGURE 5:**
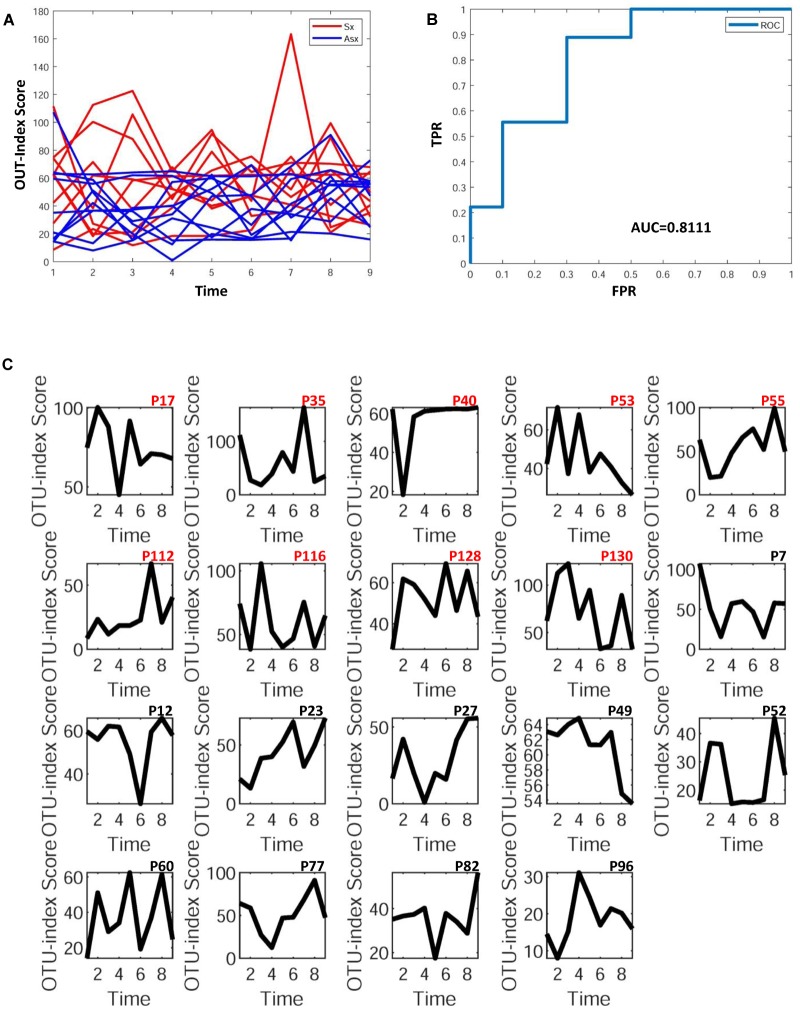
The disease classification performance of OTU markers on the bacteria in vaginal samples. **(A)** The predictive score of OTU markers based on CI index from DNB theory. The score curves of subjects from Sx BV (Sx) group are in red, and those of subjects from Asx BV and healthy (Asx) group are in blue. Generally, the Sx individuals tend to have larger scores during disease occurrence. **(B)** The classification evaluation of OTU marker score by ROC and AUC. **(C)** The OTU marker scores correspond to each subject.

Similar to the above case, the samples at the first time points of all individuals were used as the reference group. After selecting OTUs (non-zero percent > 0.5), we could calculate the sPCC (with mean and variance from the reference group) for each sample. Due to the limited number of bacteria in this data, we focused on the edges with strong correlations and finally determined the 10 strongest relations at each time point. Then, these pre-selected edges are used as the background “nodes” for constructing the edge-network and capturing the significant signal peaks of edge-biomarkers for each subject. As shown in Figure 5, to explore whether bacteria could be predictive of the eventual outcome as BV or not, we again simply used the OTU-index scores to divide individuals into Sx (BV) and Asx (not BV) groups. A threshold optimal cutoff led the single OTU-index score to achieve an AUC larger than 0.8, which means these predictors are efficient.

We also checked the individual-specific biomarkers by combining all OTUs detected on each sample for the same subject. Finally, we found 3 OTU markers in nine Sx (BV) individuals—*Aerococcus christensenii*, *Veillonellaceae*, and *Bacteria*. Meanwhile, in the Asx group (6 ABV and 4 healthy) the common markers were *Gardnerella vaginalis*, *Aerococcus christensenii*, and *Bacteria*. In order to observe more OTU markers distinguishing the two groups, we reduced the selection conditions, and 13 markers appeared in more than a half of the Sx members while 9 markers appeared in more than half of the Asx members. The Sx-special OUT markers were *Lactobacillus iners*, *BVAB2*, *Bifidobacteriaceae*, *Parvimonas micra*, and most of them have been reported in previous works (Pop et al., 2016) or are BV-associated. These results indicate again the biological significance of our selected OTU markers.

## Conclusion

There is growing interest in bolstering resistance to infections or diseases by altering the microbiota (Jia et al., 2008; Holmes, 2016; Waterman et al., 2016; Delzenne and Bindels, 2018). Here, we have presented a computational framework, i.e., iENA, to identify the key OTU features to distinguish normal and disease states, by extracting higher-order statistics and dynamic information from 16S rRNA (ribosomal RNA) gene sequencing data in a one-sample manner. As a proof-of-concept study, we carried out iENA on the temporal development data of twelve subjects (healthy adults) undergoing a challenge with intestinal microbiota by ETEC. Although the sample size is relatively small and the variations among individuals are large, our iENA achieved robust results that may lead to more confirmed conclusions. The analysis outcome from iENA indicates the following: (i) for challenged subjects, the individual symptom-related OTU markers will have stable relation (higher-order information) rather than sensitive OTU abundance; (ii) the OTU markers are significantly related to the disease development and progression (e.g., ETEC infection) which will be able to predict whether an individual would develop symptoms or not with reasonable accuracy. In addition, iENA also showed satisfactory efficiency on another dataset about BV. These consequences all demonstrate the effectiveness of iENA with DNB on an individual’s microbiota dynamics. Excluding the limitations from individual heterogeneity and sample numbers, network-based approaches like iENA will actually provide more universal tools on different types of real sequencing data (Davis-Richardson et al., 2014), which makes precision medicine more practical in clinical applications.

On account of the intestinal microbiota, iENA can explore differential microbiota pair networks based on differential OTU abundance, variance, and covariance. Although iENA has previously been validated on transcriptome datasets (Yu et al., 2017), it is also able to detect the individual-specific OTU markers on metagenomic datasets like 16S rRNA data, and disclose the higher-order associations between the microbiota and clinical symptoms during the ETEC challenge, or other disease developments like BV. Thus, the combination of new high-throughput microbiome experiments and computational systems biology approaches has the power to recommend potential candidate species in the defense against various pathogens for precision medicine.

## Author Contributions

XY and XC executed the experiment and did the data analysis. XY and ZW wrote the manuscript. XY, XC, and ZW revised the manuscript. All authors read and approved the final manuscript.

## Conflict of Interest Statement

The authors declare that the research was conducted in the absence of any commercial or financial relationships that could be construed as a potential conflict of interest.

## References

[B1] Blow N. (2008). Metagenomics: exploring unseen communities. *Nature* 453 687–690. 10.1038/453687a 18509446

[B2] Chen L. Liu R. Liu Z. P. Li M. Aihara K. (2012). Detecting early-warning signals for sudden deterioration of complex diseases by dynamical network biomarkers. *Sci. Rep.* 2:342. 10.1038/srep00342 22461973 PMC3314989

[B3] Claesson M. J. Jeffery I. B. Conde S. Power S. E. O’Connor E. M. Cusack S. (2012). Gut microbiota composition correlates with diet and health in the elderly. *Nature* 488 178–184. 10.1038/nature11319 22797518

[B4] Davis-Richardson A. G. Ardissone A. N. Dias R. Simell V. Leonard M. T. Kemppainen K. M. (2014). *Bacteroides dorei* dominates gut microbiome prior to autoimmunity in finnish children at high risk for type 1 diabetes. *Front. Microbiol.* 5:678. 10.3389/fmicb.2014.00678 25540641 PMC4261809

[B5] Delzenne N. M. Bindels L. B. (2018). Gut microbiota in 2017: contribution of gut microbiota-host cooperation to drug efficacy. *Nat. Rev. Gastroenterol. Hepatol.* 15 69–70. 10.1038/nrgastro.2017.170 29259330

[B6] Dethlefsen L. Huse S. Sogin M. L. Relman D. A. (2008). The pervasive effects of an antibiotic on the human gut microbiota, as revealed by deep 16S rRNA sequencing. *PLoS Biol.* 6:e280. 10.1371/journal.pbio.0060280 19018661 PMC2586385

[B7] Dethlefsen L. Relman D. A. (2011). Incomplete recovery and individualized responses of the human distal gut microbiota to repeated antibiotic perturbation. *Proc. Natl. Acad. Sci. U.S.A.* 108(Suppl. 1), 4554–4561. 10.1073/pnas.1000087107 20847294 PMC3063582

[B8] Forslund K. Hildebrand F. Nielsen T. Falony G. Le Chatelier E. Sunagawa S. (2015). Disentangling type 2 diabetes and metformin treatment signatures in the human gut microbiota. *Nature* 528 262–266. 10.1038/nature15766 26633628 PMC4681099

[B9] Gill S. R. Pop M. Deboy R. T. Eckburg P. B. Turnbaugh P. J. Samuel B. S. (2006). Metagenomic analysis of the human distal gut microbiome. *Science* 312 1355–1359. 10.1126/science.1124234 16741115 PMC3027896

[B10] Holmes D. (2016). Gut microbiota: antidiabetic drug treatment confounds gut dysbiosis associated with type 2 diabetes mellitus. *Nat. Rev. Endocrinol.* 12:61. 10.1038/nrendo.2015.222 26668122

[B11] Hoyles L. Fernandez-Real J. M. Federici M. Serino M. Abbott J. Charpentier J. (2018). Molecular phenomics and metagenomics of hepatic steatosis in non-diabetic obese women. *Nat. Med.* 24 1070–1080. 10.1038/s41591-018-0061-3 29942096 PMC6140997

[B12] Jernberg C. Lofmark S. Edlund C. Jansson J. K. (2010). Long-term impacts of antibiotic exposure on the human intestinal microbiota. *Microbiology* 156(Pt 11), 3216–3223. 10.1099/mic.0.040618-0 20705661

[B13] Jia W. Li H. Zhao L. Nicholson J. K. (2008). Gut microbiota: a potential new territory for drug targeting. *Nat. Rev. Drug Discov.* 7 123–129. 10.1038/nrd2505 18239669

[B14] Li M. Zeng T. Liu R. Chen L. (2014). Detecting tissue-specific early warning signals for complex diseases based on dynamical network biomarkers: study of type 2 diabetes by cross-tissue analysis. *Brief. Bioinform.* 15 229–243. 10.1093/bib/bbt027 23620135

[B15] Lozupone C. A. Stombaugh J. I. Gordon J. I. Jansson J. K. Knight R. (2012). Diversity, stability and resilience of the human gut microbiota. *Nature* 489 220–230. 10.1038/nature11550 22972295 PMC3577372

[B16] Maynard C. L. Elson C. O. Hatton R. D. Weaver C. T. (2012). Reciprocal interactions of the intestinal microbiota and immune system. *Nature* 489 231–241. 10.1038/nature11551 22972296 PMC4492337

[B17] Pop M. Paulson J. N. Chakraborty S. Astrovskaya I. Lindsay B. R. Li S. (2016). Individual-specific changes in the human gut microbiota after challenge with enterotoxigenic *Escherichia coli* and subsequent ciprofloxacin treatment. *BMC Genomics* 17:440. 10.1186/s12864-016-2777-0 27277524 PMC4898365

[B18] Pop M. Walker A. W. Paulson J. Lindsay B. Antonio M. Hossain M. A. (2014). Diarrhea in young children from low-income countries leads to large-scale alterations in intestinal microbiota composition. *Genome Biol.* 15:R76. 10.1186/gb-2014-15-6-r76 24995464 PMC4072981

[B19] Pushkarev A. Inoue K. Larom S. Flores-Uribe J. Singh M. Konno M. (2018). A distinct abundant group of microbial rhodopsins discovered using functional metagenomics. *Nature* 558 595–599. 10.1038/s41586-018-0225-9 29925949 PMC11128463

[B20] Qin J. Li Y. Cai Z. Li S. Zhu J. Zhang F. (2012). A metagenome-wide association study of gut microbiota in type 2 diabetes. *Nature* 490 55–60. 10.1038/nature11450 23023125

[B21] Rakoff-Nahoum S. Foster K. R. Comstock L. E. (2016). The evolution of cooperation within the gut microbiota. *Nature* 533 255–259. 10.1038/nature17626 27111508 PMC4978124

[B22] Ravel J. Brotman R. M. Gajer P. Ma B. Nandy M. Fadrosh D. W. (2013). Daily temporal dynamics of vaginal microbiota before, during and after episodes of bacterial vaginosis. *Microbiome* 1:29. 10.1186/2049-2618-1-29 24451163 PMC3968321

[B23] Round J. L. Mazmanian S. K. (2009). The gut microbiota shapes intestinal immune responses during health and disease. *Nat. Rev. Immunol.* 9 313–323. 10.1038/nri2515 19343057 PMC4095778

[B24] Sung J. Kim S. Cabatbat J. J. T. Jang S. Jin Y. S. Jung G. Y. (2017). Global metabolic interaction network of the human gut microbiota for context-specific community-scale analysis. *Nat. Commun.* 8:15393. 10.1038/ncomms15393 28585563 PMC5467172

[B25] Vedoy O. B. Hanevik K. Sakkestad S. T. Sommerfelt H. Steinsland H. (2018). Proliferation of enterotoxigenic *Escherichia coli* strain TW11681 in stools of experimentally infected human volunteers. *Gut Pathog.* 10:46. 10.1186/s13099-018-0273-6 30349586 PMC6192177

[B26] Wagner J. Chelaru F. Kancherla J. Paulson J. N. Zhang A. Felix V. (2018). Metaviz: interactive statistical and visual analysis of metagenomic data. *Nucleic Acids Res.* 46 2777–2787. 10.1093/nar/gky136 29529268 PMC5887897

[B27] Wang H. Li Y. Feng X. Li Y. Wang W. Qiu C. (2016). Dysfunctional gut microbiota and relative co-abundance network in infantile eczema. *Gut Pathog.* 8:36. 10.1186/s13099-016-0118-0 27453732 PMC4957860

[B28] Wang L. Yu X. Zhang C. Zeng T. (2018). Detecting personalized determinates during drug treatment from omics big data. *Curr. Pharm. Des.* 24 3727–3738. 10.2174/1381612824666181106102111 30398110

[B29] Waterman C. Calcul L. Beau J. Ma W. S. Lebar M. D. von Salm J. L. (2016). Miniaturized Cultivation of Microbiota for Antimalarial Drug Discovery. *Med. Res. Rev.* 36 144–168. 10.1002/med.21335 25545963

[B30] Wu G. D. Chen J. Hoffmann C. Bittinger K. Chen Y. Y. Keilbaugh S. A. (2011). Linking long-term dietary patterns with gut microbial enterotypes. *Science* 334 105–108. 10.1126/science.1208344 21885731 PMC3368382

[B31] Yu X. Zeng T. Wang X. Li G. Chen L. (2015). Unravelling personalized dysfunctional gene network of complex diseases based on differential network model. *J. Transl. Med.* 13:189. 10.1186/s12967-015-0546-5 26070628 PMC4467679

[B32] Yu X. Zhang J. Sun S. Zhou X. Zeng T. Chen L. (2017). Individual-specific edge-network analysis for disease prediction. *Nucleic Acids Res.* 45:e170. 10.1093/nar/gkx787 28981699 PMC5714249

[B33] Yu X. T. Zeng T. (2018). Integrative analysis of omics big data. *Methods Mol. Biol.* 1754 109–135. 10.1007/978-1-4939-7717-8_7 29536440

[B34] Zeng T. Sun S. Y. Wang Y. Zhu H. Chen L. (2013). Network biomarkers reveal dysfunctional gene regulations during disease progression. *FEBS J.* 280 5682–5695. 10.1111/febs.12536 24107168

[B35] Zeng T. Wang D. C. Wang X. Xu F. Chen L. (2014). Prediction of dynamical drug sensitivity and resistance by module network rewiring-analysis based on transcriptional profiling. *Drug Resist. Updat.* 17 64–76. 10.1016/j.drup.2014.08.002 25156319

[B36] Zeng T. Zhang W. Yu X. Liu X. Li M. Chen L. (2016). Big-data-based edge biomarkers: study on dynamical drug sensitivity and resistance in individuals. *Brief. Bioinform.* 17 576–592. 10.1093/bib/bbv078 26411472

